# Identification of VASH1 as a Potential Prognostic Biomarker of Lower-Grade Glioma by Quantitative Proteomics and Experimental Verification

**DOI:** 10.1155/2022/2621969

**Published:** 2022-11-30

**Authors:** Yirizhati Aili, Nuersimanguli Maimaitiming, Aierpati Maimaiti, Wen Liu, Hu Qin, Wenyu Ji, Yusufu Mahemuti, Yongxin Wang, Zengliang Wang

**Affiliations:** ^1^Department of Neurosurgery, The First Affiliated Hospital of Xinjiang Medical University, Urumqi, Xinjiang, China; ^2^Department of Oncology, The First Affiliated Hospital of Xinjiang Medical University, Urumqi, Xinjiang, China

## Abstract

**Background:**

VASH1 is a novel angiogenic regulatory factor, that participates in the process of carcinogenesis and the development of diverse tumors. Our study aimed to investigate the expression and prognostic value of the VASH1 in Lower-Grade Glioma (LGG), to explore its functional network in LGG and its effects on biological behaviors.

**Methods:**

LGG transcriptome data, somatic mutation profiles and clinical features analyzed in the present study were obtained from the TCGA, GTEx, CCLE, CGGA, UALCAN, and GEPIA2 databases, as well as clinical data and tissue sections of 83 LGG patients in our hospital. The expression characteristics of VASH1 in LGG were investigated by univariate, multivariate, immunohistochemistry, qRT-PCR, and western-blot. Subsequently, we analyzed the prognostic significance of VASH1 in LGG patients by survival analysis, subject operation characteristic curve, correlation analysis, external validation, independent prognostic significance analysis, and clinical stratification, and confirmed its biological effect on glioma cell lines in vitro. Finally, we performed GO, KEGG, and GSEA to clarify biological functions and related pathways. CIBERSORT and ESTIMATE algorithms were used to calculate the proportion of immune cells and immune microenvironment fraction in LGG.

**Result:**

We found that VASH1 is highly expressed in LGG tissues and is associated with poor prognosis, WHO grade, IDH1 wild-type, and progressive disease (*P* < 0.05). Multivariate and the Nomogram model showed that high VASH1 expression was an independent risk factor for glioma prognosis and had better prognostic prediction efficacy in different LGG Patient cohorts (HR = 4.753 and *P*=0.002). In vitro experiments showed that knockdown of VASH1 expression in glioma cell lines caused increased glioma cell proliferation, invasion, and migration capacity. The mechanism may be related to VASH1 promoting microtubule formation and remodeling of immune microenvironment.

**Conclusion:**

Our study firstly found that high VASH1 expression was associated with poor prognosis. In addition, We identified the possible mechanism by which VASH1 functioned in LGG. VASH1 inhibits the invasion and migration of tumor cells by affecting microtubule formation and immune infiltration in the tumor microenvironment. May be an important endogenous anti-tumor factor for LGG and provide a potential biomarker for individualized treatment of LGG.

## 1. Introduction

Glioma is a common primary tumor of the central nervous system in adults. In 2016, the World Health Organization (WHO) classified glioma into grade I∼IV according to its malignant degree and pathological characteristics. The higher the grade, the higher the malignant degree, and the worse the prognosis [1]. WHOII and WHOIII gliomas are defined as lower-grade Glioma (LGG) due to their different origin of glioblastoma (GBM), and LGG accounts for 43.2% of primary intracranial gliomas [2]. Studies have shown that although LGG has a lower recurrence rate compared to GBM, but the recurrence rate varies between patients, some even reaching 80–90%, and 16.1–21% of LGG patients develop malignancy with a survival time of only 7 years [3]. Therefore, identifying and screening high-risk LGG with the potential for deterioration is an important task to be urgently solved.

As genetic testing techniques continue to develop and mature, researchers have found that LGG molecular signatures can further guide risk stratification. In recent years, it has been found that the progress of LGG is closely related to some gene phenotypes, which directly regulate the malignant phenotype of LGG. For example, Isocitrate dehydrogenase (IDH) mutation, deletion of the short arm of chromosome 1, and the long arm of chromosome 19 (1 p/19 q), TP53 mutation, MGMT methylation status, TERT promoter mutation, CDKN2A/B deletion, and EGFR amplification. [4]. Therefore, the importance of glioma genotype-phenotype was emphasized in the WHO Classification of Neurological Tumors in 2021, opening a new concept of glioma diagnosis in the genetic era [5]. This further encourages us to explore and discover biomarkers closely related to LGG, more accurately distinguish and screen high-risk LGG patients, and early intervention to improve their overall survival.

VASH1 belongs to a class of angiogenic regulatory proteins in the family of angiogenesis inhibitors that negatively regulate angiogenic endothelial cell-derived factors [6]. Recent studies have shown that VASH1 can be selectively expressed not only in endothelial cells but also in tumor cells and some immune cells, participating in the occurrence and development of tumors and immune responses [7, 8]. With the continuous progress of research on VASH1, more and more scholars have developed the special biological characteristics of VASH1 and applied them to clinical treatment as a tumor target. At present, much literature has discussed the clinical role of VASH1 in gastric cancer [9], ovarian cancer [10], colorectal cancer [11], esophageal cancer [12], prostate cancer [13], and nonsmall cell lung cancer [14]. In LGG, there are few reports on the role of VASH1. Further study on the molecular mechanism of VASH1 affecting the genesis and development of LGG will help to identify molecular targets for tumor therapy and provide clues for the development of new and more powerful antitumor tools.

In this study, we confirmed the correlation between VASH1 and LGG tissue expression, WHO grade, IDH1 mutation, tumor mutation burden (TMB), immune cell infiltration, and prognosis by using TCGA, GTEx, CCLE, CGGA, UALCAN, GEPIA2, and Timer databases. In addition, the expression of VASH1 in LGG tissues was confirmed by immunohistochemistry, western-blot, and qRT-PCR, and the correlation risk model of VASH1 was established by Kaplan-Meier survival analysis and multivariate Cox analysis, and the accuracy of the model was verified by using a nomogram, C-index, and AUC curve. Meanwhile, the biological effects of VASH1 on glioma cells were confirmed by in vitro experiments.

## 2. Method and Materials

### 2.1. Data Collection and Preprocessing

From the cancer genome Atlas (TCGA) database (https://portal.gdc.cancer.gov/) and genotype-organization express project (GTEx) database (https://gtexportal.org/), we downloaded 33 kinds of tumor gene expression data and normal tissue and tumor tissue clinical information. Transcriptome (TPM), somatic mutation data, copy number variation (CNV), and clinical phenotype data for LGG were downloaded from the TCGA database. Corresponding to heavy annotation in gene chip RNA probe, we download the appropriate RNA genome sequence information and data from the GENECODE database (https://www.gencodegenes.org/human/HYPERLINK “https://www.gencodegenes.org/human/“ \o “https://www.gencodegenes.org/human/“https://www.gencodegenes.org/human/). The RNA expression profile of the reannotated microarray was constructed by matching the sequence information of the probe with that of RNA. The human genome annotation file (GRCh38/hg38) from the UCSC database (http://hgdownload.cse.ucsc.edu/HYPERLINK “http://hgdownload.cse.ucsc.edu/“ \o “http://hgdownload.cse.ucsc.edu/“http://hgdownload.cse.ucsc.edu/) to download. In addition, the department of cancer cells encyclopedia (CCLE) database downloaded 21 tumor cell lines (such as breast, thyroid, and uterine) information (https://portals.broadinstitute.org/). Finally, based on the FTO expression levels of 33 cancers, univariate survival analysis was used to study the prognosis of patients in terms of overall survival (OS) and disease-specific survival (DSS). Kaplan-Meier curves and forest maps were visualized for cancer with significant statistical differences.

### 2.2. Correlation Between NCAPG Expression and Clinical Features in Glioma

The correlations between VASH1 expression and various clinical characteristics were evaluated using the Xiantaoxueshu database (https://www.xiantao.love/writings). Clinical features evaluated included World Health Organization (WHO) tumor grades, deletion of sequences at chromosomes 1 p and 19 q, mutations in the gene encoding isocitrate dehydrogenase (IDH), patient age, and responses to radiotherapy and chemotherapy.

### 2.3. Correlation Between Tumor Immune Cell Infiltration and VASH1 Gene Expression

Tumor immune to assess resource (TIMER, https://cistrome.shinyapps.io/timer/) is a comprehensive database, that can be systematically analyzed of various types of cancer of the immune-infiltrating [15]. Spearman correlation was used to estimate the correlation between VASH1 expression and levels of 47 immune checkpoint genes in tumor immune-infiltrating cells (CD4^+^T cells, B cells, CD8^+^T cells, macrophages, neutrophils, and dendritic cells) in 33 cancers. In addition, association analysis of VASH1 with stroma scores for multiple cancers was evaluated by software estimation. At the same time, the relationship between gene expression and the immune score was analyzed in 33 tumor samples using the R package ESTIMATE. Secondly, TMB is defined as the total number of somatic gene coding mutations existing in tumor tissues, such as deletion errors or gene insertions [16]. MSI refers to a strongly mutated phenotype caused by loss of DNA mismatch repair activity [17]. Both TMB and MSI are potential predictive biomarkers of immune checkpoint therapy. We extracted TMB and MSI data from the TCGA database. Spearman analysis was used to estimate the correlation between VASH1 expression level and TMB or MSI status.

### 2.4. VASH1-Related Gene Enrichment Analysis

We first searched the STRING (https://string-db.org/) [18] and GeneMANIA (https://genemania.org/) [19] website using the query of a single protein name (“VASH1”) and organism (“Homo sapiens”). Subsequently, we set the following main parameters: minimum required interaction score (“Low confidence (0.150)”), meaning of network edges (“evidence”), max number of interactors to show (“not more than 50 interactors” in 1st shell) and active interaction sources (“experiments”). Finally, the available experimentally determined VASH1-binding proteins were obtained.

### 2.5. GO Annotation and KEGG Pathway Enrichment Analysis

Differentially expressed genes (DEGs) between two groups were screened by using the “DESeq2” package in R software according to the thresholds of |log2FoldChange|>1 and adjusted *P* < 0.05. Gene Ontology (GO) and Kyoto Encyclopedia of Genes and Genomes (KEGG) enrichment analyses were performed to annotate the biological functions of DEGs and VASH1-related genes through “clusterProfiler” package. With the annotated gene sets in “h.all.v7.4.symbols.gmt” chosen as the reference gene sets, gene set enrichment analysis (GSEA) was conducted to investigate the potential regulatory mechanisms of VASH1.

### 2.6. External Validation of Genes in the VASH1 mRNA Risk Score Model

The feature model genes were verified by CGGA mRNA seq-693 and CGGA mRNA seq-325 in the Chinese Glioma Genome Atlas (CGGA) database and GSE16011 in the Gene Expression Summary Database (GEO). The same formula was used to calculate the risk score, and Boxplot was used to compare the gene expression of different genders, tumor stage (II and III), tumor types (primary and recurrent), and VASH1 expression status.

### 2.7. Analysis of the Correlation Between the VASH1 Expression and Drug Sensitivity

We utilized the GDSC (https://www.cancerrxgene.org/) and CTRP (http://portals.broadinstitute.org/ctrp.v2.1/) databases to analyze the correlation between VASH1 expression and drug sensitivity.

### 2.8. Management of Tissue Specimens

All LGG patients and tissue samples involved in this study were from the neurosurgery sample bank of the First Affiliated Hospital of Xinjiang Medical University. 204 cases of intracranial tumor resection in the First Affiliated Hospital of Xinjiang Medical University from January 2013 to December 2019 were randomly selected. Excluding meningioma, high-grade glioma, WHOI glioma, patients who had received preoperative radiotherapy and chemotherapy, and patients with incomplete follow-up information, the remaining 83 LGG cases and 25 precancerous tissue samples were paraffin-embedded for immunohistochemical staining. Postoperative intracranial tumors were independently diagnosed as LGG by 2 pathologists according to WHO grading standards, including 52 WHOII cases and 31 WHOIII cases. Basic clinical data of 83 patients were collected and standard clinical follow-up was performed. The follow-up period of the study was up to the end of December 2020.

The 8 pairs of lower-grade glioma tissues and corresponding precancerous tissues used for real-time PCR were obtained from intraoperative specimens of glioma patients undergoing surgical treatment in the First Affiliated Hospital of Xinjiang Medical University. Inclusion criteria: according to WHO grading standards, WHO grade II and WHO Grade III glioma patients were diagnosed by pathology in our hospital. None of the patients had received radiotherapy or chemotherapy before surgery. Each patient was studied with tumor tissue and corresponding para cancer tissue. After surgery, the tissue was immediately put into liquid nitrogen and then stored in a refrigerator at −80°C. All patients signed the informed consent. According to the Declaration of Helsinki, the samples and case data used in this study were approved by the Ethics Committee of the First Affiliated Hospital of Xinjiang Medical University.

### 2.9. The Expression of VASH1 Was Detected by Immunohistochemistry

Immunohistochemical analysis, LGG pathological tissue was embedded in paraffin and sectioned on a 4 *μ*m microtome. Slices were placed on slides and dehydrated with different concentrations of alcohol solutions (75%, 80%, 90%, 95%, and 100%) at different times and cleaned with xylene. A two-step indirect immunohistochemical staining was used in this study. Rabbit antibodies against human VASH1 (Abcam, United Kingdom) were diluted to 1 : 200 and 1 : 250, respectively. Antibody staining was performed overnight at 4°C. The reaction of 3,3′-Diaminobenzoaniline (DAB) substrate-chromogen with peroxidase-conjugated secondary antibody was used to fix paraffin-embedded tissue sections with formalin. DAB can react with the slices to produce brown products insoluble in ethanol and xylene at the antigen site. The sections were rinsed with phosphate buffer solution 3 times, and then the expression of target proteins in the tissues was observed with a light microscope at 200 times magnification. Immunohistochemical results were evaluated by two independent pathologists of our hospital. Immunohistochemical staining intensity was classified according to the following standards: no staining = 0 points, mild staining = 1 point, moderate staining = 2 points, and severe staining = 3 points. Staining area scoring criteria were as follows: positive staining percentage was 0% (0 points), 1–25% (1 points), 26–50% (2 points), 56–75% (3 points), and >75% (4 points). Final score = staining degree score×staining area score. Samples with a score of <6 were considered to have low expression of VASH1, and those with a score of≥6 were considered to have high expression of VASH1.

They were divided into the VASH1 high expression group and VASH1 low expression group. Finally, we divided LGG patients into high-risk groups and low-risk groups according to the median value of VASH1. The Kaplan-Meier method was used to draw survival curves of the two groups to predict their prognostic significance in OS and DFS, and *P* < 0.05 indicated a significant statistical difference.

### 2.10. Cell Line and Cell Culture

All cell lines including U-87, U-251, and A-172 were purchased from the Cell Bank of the Chinese Academy of Sciences (China). All cell lines were cultured in DMEM medium with 10% newborn bovine serum, supplemented with penicillin 100*μ*/mL and streptomycin 100 *μ*g/mL, in a 5% CO_2_ incubator with saturated humidity and 37°C constant temperature.

### 2.11. RNA Extraction and qRT-PCR

**Figure 1:**
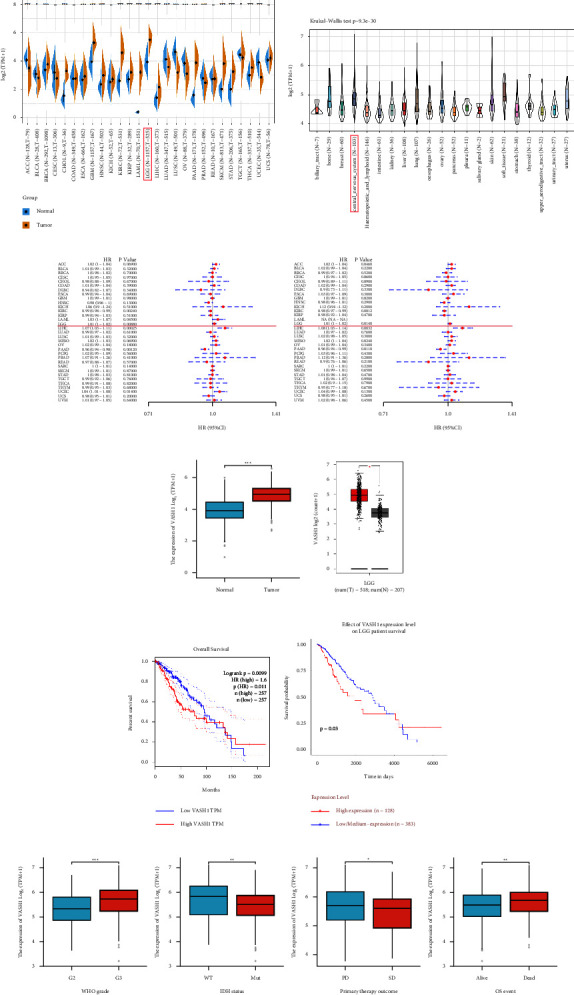
Abnormal expression of VASH1 in generalized carcinoma. (a) Expression differences of VASH1 in 27 cancer types integrated from GTEx and TCGA databases. (b) VASH1 expression levels in 21 tumor cells from the CCLE database. (c) Forest diagram of the relationship between VASH1 expression and OS in 33 tumors. (d) Forest diagram of the relationship between VASH1 expression and DSS in 33 cancers. (e),e mRNA expression levels of RPL4P4 in different tumor tissues and normal tissues analyzed by TCGA and GTEx. (f) VASH1 expression is signi:cantly up-regulated in glioma (GPEIA2). (g and h) Kaplan-Meier survival analysis of VASH1 expression in LGG patient of GPEIA2 (g) and UALCAN (h). (I),e correlation between VASH1 expression and different clinical features based TCGA-LGG patient.

Total RNA was extracted from tissues and cells according to TRIzol reagent instructions, and cDNA reverse transcription was performed according to the instructions of the RT-PCR kit (Invitrogen, United States). GAPDH and VASH1 expression levels were detected by qRT-PCR using SYBR Green qPCR Master Mix (High ROX) (Servicebio, Wuhan, China). Results: the expression level of GAPDH was taken as standard. The PCR primer sequence was designed and synthesized by Servicebio (Wuhan). Primer information is shown in Figure 1(a), The relative expression levels of VASH1 were quantitatively calculated by the 2^(−ΔΔCT)^ method. The amplification reaction included the following steps: Predenaturation at 95°C for 10 min, denaturation at 95°C for 15 s over 40 cycles, and extension at 60°C for 30 s. From 65°C to 95°C, the fluorescence signal was collected every 0.3°C.

### 2.12. Western-Blot

Collecting cells, cell lysis solution treatment, the supernatant, centrifuge after Coomassie brilliant blue method to determine protein concentration, polypropylene phthalic amide gel electrophoresis, The protein electricity is transferred to the nitrate fiber membrane, combined with VASH1 antibody (1 : 10 dilution degrees 00, Abcam), combined with horseradish peroxide enzyme after two combinations, ECL method after color photograph. Finally, the gray values of each band were determined by Image-Pro Plus 4.5 Image analysis system to reflect the expression level of VASH1 protein.

### 2.13. Construction and Transfection Of Lentivirus

The relevant information of the VASH1 gene was searched through the Genbank database, and the online design software of Ambion was applied to select the human VASH1 gene (gene serial number): The sequence of siRNA was 5′-CGACCGGAAGAAGGATGTTTC-3′ at 1307–1331 of cDNA. BLAST search confirmed no homology with known human gene sequences other than VASH1. DNA Oligo of VASH1 shRNA was designed and synthesized, double-stranded DNA was formed by annealing, HpaI and EcoRI were digested and ligated, and transformed into PGCL-GFP expression plasmid of E. coli DH5a. Recombinant positive clones were selected for PCR and sent for sequencing identification (Shanghai Genechem Technology). 293T cells were cotransfected with pgCL-GFP vector 20 *μ*g, pHelper 1.0 vector 15 *μ*g, and pHelper 2.0 vector 10 *μ*g. 8 h after transfection, the cells were replaced with a complete culture medium. After 48 h of culture, cell supernatant rich in lentivirus particles was collected, and was stored at −80°C for future use.

### 2.14. Transwell Assay

Transwell cell was placed in a 24-well plate, the substrate glue was added to the Transwell cell, and the complete culture medium was added to the substrate. After digestion and resuspension, the cells of each group were inoculated in the upper chamber of Transwell, and the number of cells was 3 × 10^4^. After 48 h culture, the cells that did not invade the subchamber were washed away. Then, it was fixed with 4% polymethanol and stained with 0.1% crystal violet for 20 min. The number of cells invading the subcompartment in each field was counted under an inverted microscope.

### 2.15. Wound*-*Healing Assay

The cells in each group were digested by trypsin and inoculated into 6-well plates with 1 × 10^6^ cells in each well. Then, the cells were cultured at 37°C, 5% CO_2_, and 100% relative humidity until the cells reached about 90% confluence. Then, scratches were made from top to bottom with 200 *μ*L pipette tip and the scratched cells were washed away with PBS buffer. Then, the culture was continued for 24 h under the same conditions. The single-layer images were observed by an inverted microscope and the migration ability of cells was analyzed by measuring the movement distance of the cell front and the width of the scratch.

### 2.16. Statistical Analysis

All bioinformatics and clinical characters analyses were performed in R version 4.0.3, and all experimental data analysis was carried out in GraphPad Prism 9 and SPSS 20.0. The significance of the differences between the groups was assessed by the Student's *t*-test. The Chi-square test or Fisher test was used for categorical variables, and the Wilcoxon test was used for continuous data. Survival differences were calculated using Kaplan-Meier and logarithmic rank tests. In addition, the use of interactive gene expression profile analysis GEPIA2 (http://gepia2.cancer-pku.cn/) and UALCAN (http://ualcan.path.uab.edu/index.html) [20]. Different expression analyses were further performed on LGG samples from TCGA and normal samples from matched TCGA normal and genotype-tissue expression (GTEx) data. *P* < 0.05 was statistically significant.

## 3. Result

### 3.1. Expression and Prognostic Potential of VASH1 in Pan-Cancer

**Figure 2:**
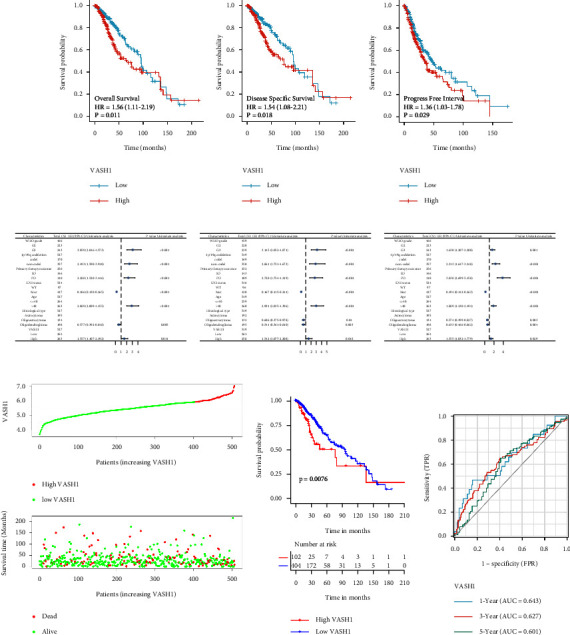
The prognosis of RPL4P4 in glioma examine by TCGA databases. (D–F),eforest plot indicated that the prognosis in the presence of OS (D), DSS (E), and PFI (F). (G) Comparison of VASH1 expression with survival time and survival status. (H) Di9erence of VASH1 expression risk group model in LGG patient. (I) ROC analysis showing the predictive value of VASH1 in LGG based on TCGA.

First, we integrated the expression of VASH1 in cancer and normal tissue samples from the GTEx and TCGA databases and found that compared with the GTEx normal control group, significantly higher VASH1 was expressed in various types of cancer, including ACC, BLCA, BRCA, CHOL, ESCA, GBM, HNSC, KIRC, KIRP, LAML, LGG, LIHC, LUAD, LUSC, OV, PAAD, PRAD, SKCM, STAD, TGCT, THCA, and UCEC (Figure 2(a), Table 1). Meanwhile, we used the CCLE database, the results showed that VASH1 expression was elevated in different glioma cell lines (Figure 2(b)). In brief, the present results suggest that VASH1 is differentially expressed in multiple cancers. To explore the relationship between VASH1 and the clinical outcome in patients with 33 cancers, a univariate analysis was performed using the dataset of TCGA. Forest plots showed that VASH1 had significant effects on the survival time of specific tumor types, with VASH1 associated with OS and DSS in LGG patients (*P*=0.008 and*P*=0.014) (Figures 2(c) and 2(d)).

For further exploration, We using TCGA and GEPIA online analysis website, the results showed that VASH1 was highly expressed in LGG tissues (Figures 2(e) and 2(f)), and through K–M survival analysis we found that VASH1 high expression correlated with shorter overall survival in LGG patients (Figures 2(g) and 2(h)). It was found to be associated with the clinical features of LGG (Table 2). We found that VASH1 high expression correlates with WHO grading, IDH1 mutation status, primary therapy outcome, and survival time (Figure 2(i)).

### 3.2. Correlation Between VASH1 Expression and Clinical Outcomes in LGG Patients

**Figure 3:**
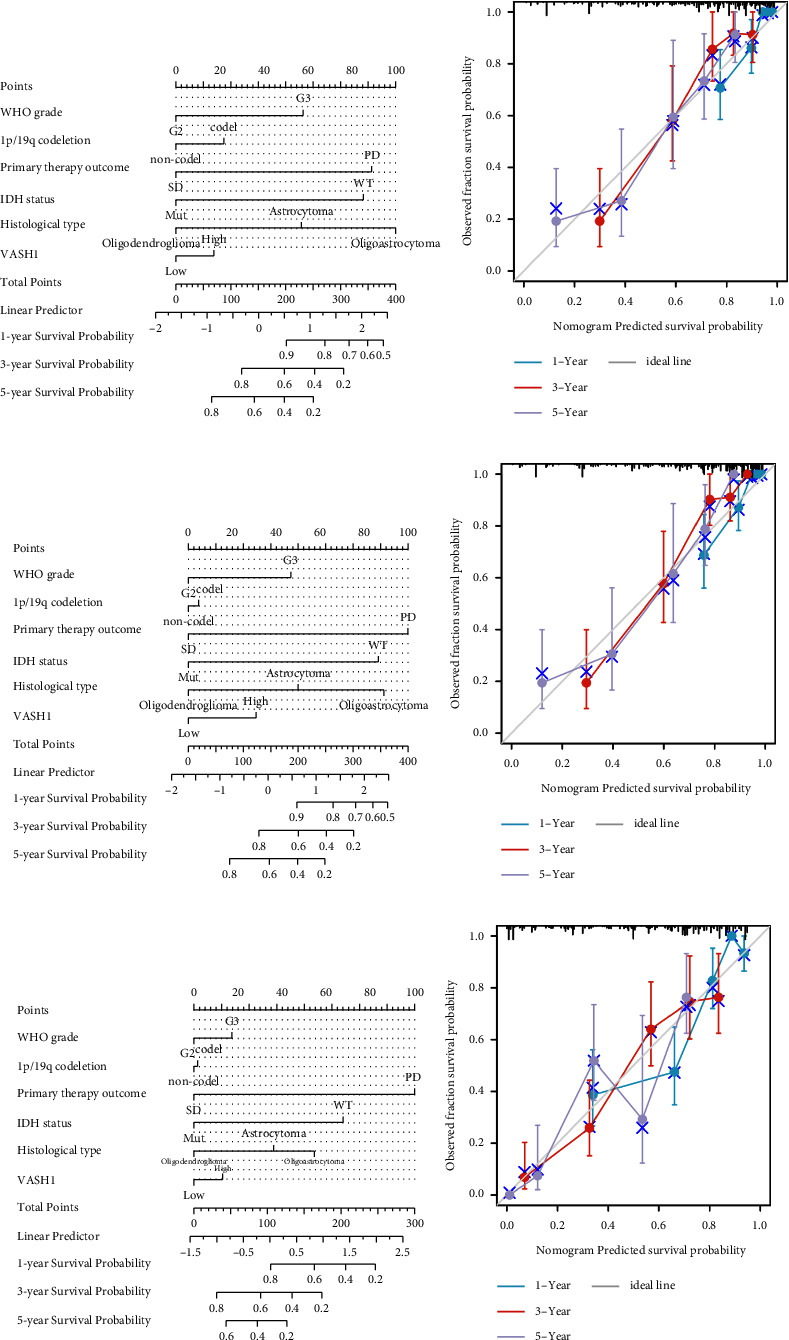
Construction a nomogram to predict the prognosis of VASH1 in LGG. Construction of a nomogram to predict the OS (a), DSS (c), and PFI (e) in patients with LGG. ,e calibration curve used to display the TCGA-LGG cohort for OS (b), DSS (d), and PFI (f).

Based on the median VASH1 expression in the samples in the database, patients were divided into VASH1 high expression and low expression groups. The high expression of VASH1 was found to correlate with poor OS (Figure 3(a)), DSS (Figure 3(b)), and PFI (Figure 3(c)), and a visual forest plot was plotted (Figures 3(d), 3(e), 3(f). Univariate results showed that high expression of VASH1 in gliomas was significantly associated with poor LGG prognosis (Table 3). Subsequently, we established a risk model based on VASH1 expression, and the risk factor plot results showed that the mortality rate increased with the increase of VASH1 expression (Figure 3(g)), and the K–M survival analysis of the model found that the mortality rate of LGG patients in the VASH1 high expression group was higher (Figure 3(h)), and the prognostic prediction efficacy of the model at 1, 3, and 5 years had good accuracy (Figure 3(i)). The above results further confirm that the increase in VASH1 expression is closely related to the occurrence and development of LGG.

**Figure 4:**
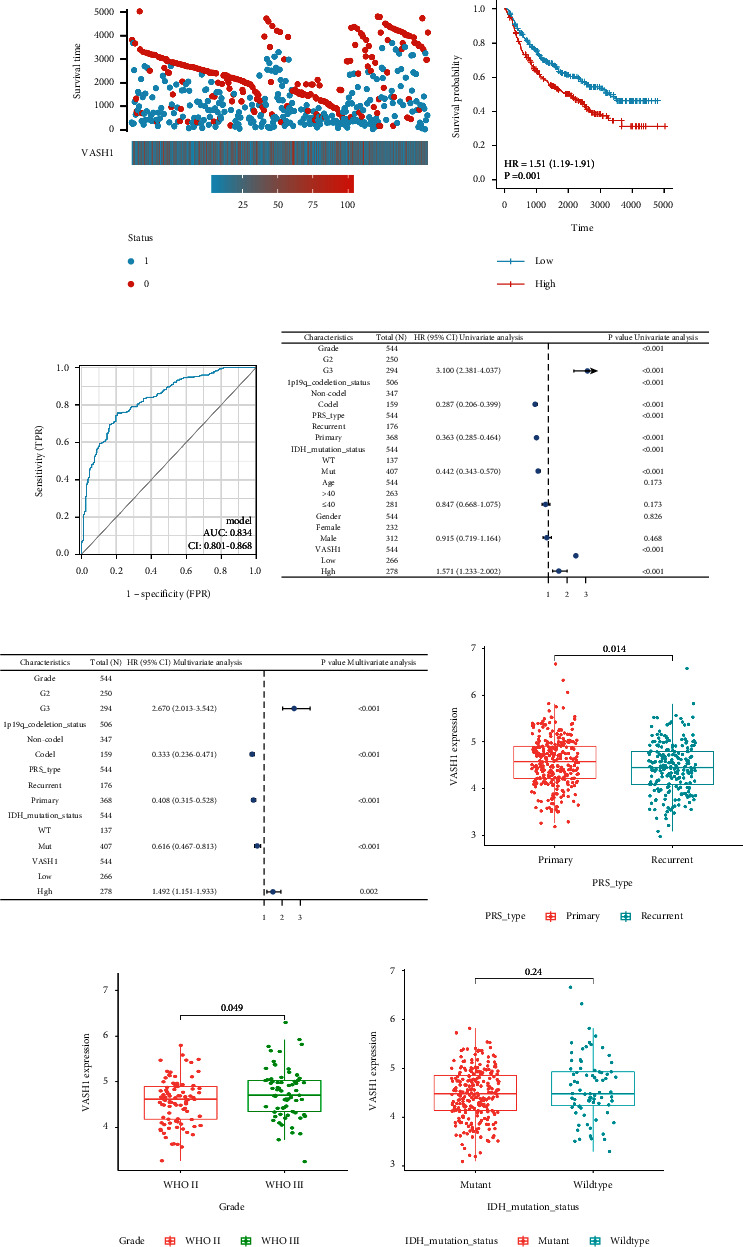
Evaluation of the performance of the VASH1 using CGGA datasets. (a) Comparison of VASH1 expression with survival time and survival status. (b) Prognostic value of VASH1 in LGG patient. (c) ROC analysis showing the predictive value of VASH1 in LGG based on CCGA. (d and e) Univariate and multivariate analysis based on CGGA data. (f–h) ,e correlation between VASH1 expression and di9erent clinical features based CCGA LGG patient.

The ability of a nomogram that included VASH1 expression, histological type, sex, IDH mutant status, WHO grade, chromosome 1 p/19 q codeletion, and primary therapy outcome to accurately predict prognosis in LGG patients was tested. This nomogram was found to predict 1-, 3-, and 5-year OS, DSS, and PFI in patients with LGG (Figures 4(a)–4(f).

### 3.3. The Correlation of VASH1 and LGG was Further Verified By An External Database

**Figure 5:**
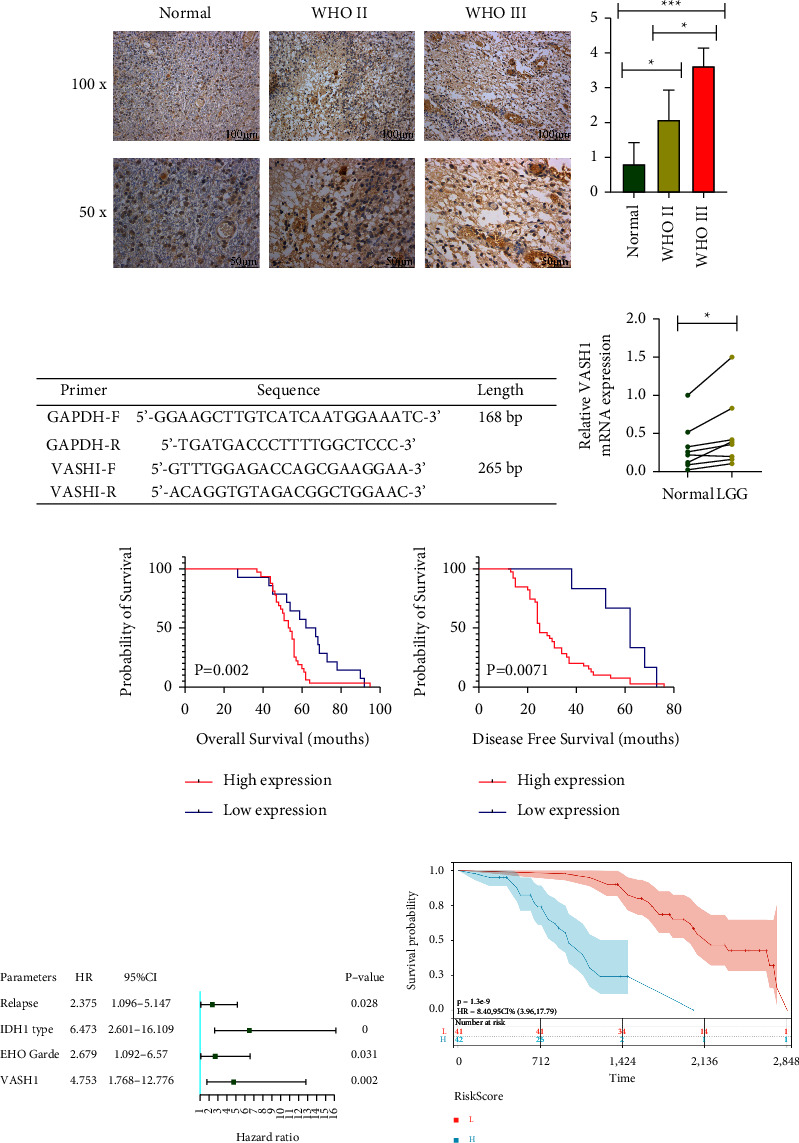
Validation of the elevated expression and prognostic value of VASH1 based on our LGG cohort. (a) Representative immunohistochemistry staining of VASH1 in WHOII, WHOIII, and adjacent normal tissues. (b) Quanti:ed data of the score for VASH1 staining. (c d) qRT-PCR was used to con:rm the expression level of LGG and adjacent normal tissues. (e) OS curves of patients in the LGG strati:ed by VASH1 expression levels (our cohort). (f) DFS curves of patients in the LGG strati:ed by VASH1 expression levels (our cohort). (g) Multivariate analysis based on our LGG data. (h) ,e K-M survival analysis was performed, strati:ed by the LGG-independent risk factors.

To further validate VASH1 expression levels and the clinical prognosis of LGG in other independent datasets on different platforms. First, we stratified LGG patients according to VASH1 expression and found that high VASH1 expression was positively correlated with LGG mortality (Figure 5(a)) and associated with shorter survival (Figure 5(b)). At the same time, the model is further verified by ROC curve, and it is found that the model has good accuracy(AUC = 0.834) (Figure 5(c)). Subsequently, we verified the correlation between VASH1 and LGG patient prognosis by univariate and multivariate analysis (Table 3) and plotted a visual forest map (Figures 5(d) and 5(e)), confirming that VASH1 high expression is an independent risk factor for LGG patient prognosis. Finally, we validated the relationship between VASH1 and various clinical features of LGG and found that it was closely related to WHO grades (Figure 5(f)) and recurrent (Figure 5(g)), but there was no significant correlation with IDH types (Figure 5(h)), which was inconsistent with the results in the TCG database, possibly due to differences in the ethnicity, number, and pathological type of LGG patients.

### 3.4. The Expression of VASH1 in our LGG Cohort

Next, we took immunohistochemistry to detect VASH1 expression in our 83 LGG patients tissues. According to the observations of two pathologists (unknown patient information), immunohistochemical results showed that VASH1 protein was mainly brown positive in the nucleus and cytoplasm of glioma cells and endothelial cells (Figure 1(a)). Meanwhile, VASH1 was positively expressed in most LGG tissues (53/83, 63.9%), and its expression level also increased with increasing malignancy, and the difference was statistically significant (*P* < 0.05) (Figure 1(b)). Subsequently, in order to continue to explore the expression of VASH1 in fresh frozen LGG tissues. The results showed that VASH1 mRNA expression levels were significantly higher in all LGG fresh tissue samples than in the corresponding peritumoral tissues (*P* < 0.01) (Figure 1(c) and 1(d)).

### 3.5. Relationship Between VASH1 Expression, Pathological Parameters and Prognosis in LGG Patients

Based on the expression level of VASH1, we analyzed the clinicopathological parameters and prognostic factors in 83 LGG patients in our hospital, and we found that VASH1 expression was not significantly compared with the age, sex, pathological type, tumor size, site, epilepsy, and KPS scores of LGG patients. However, the VASH1 expression level was correlated with tumor recurrence, WHO grade, and IDH1 wild-type expression level (*P* < 0.05) (Table 4). We found that VASH1 expression is closely related to the grade of LGG malignancy and tumor recurrence, so does the VASH1 expression level affect the prognosis of LGG patients? In this regard, we systematically followed the postoperative prognosis of each LGG patient, based on our follow-up results, we analyzed their OS and DFS, which showed that the OS and DFS of patients with high VASH1 expression group were significantly lower than those with low VASH1 expression (*P* < 0.05). K–M survival curves were drawn for Figures 1(e) and 1(f) of two survival groups.

### 3.6. Univariate and Multivariate Analysis in Our LGG Patient Cohort

We included univariate characteristics and VASH1 expression affecting the prognosis of LGG patients in the COX risk regression model in univariate and multivariate regression analysis. The univariate analysis showed that tumor recurrence, WHO grade, IDH1 wild-type, and high VASH1 expression were individual risk factors affecting the prognosis of LGG patients (Table 5). Furthermore, multivariate analysis showed that tumor recurrence (HR = 2.375 and *P*=0.028), WHO grade (HR = 2.679 and *P*=0.031), IDH1 wild-type (HR = 6.473 and *P* < 0.001), and high VASH1 expression (HR = 4.996 and *P* < 0.002) were independent risk factors for LGG patients (Figure 1(g)) (Table 6). Meanwhile, we stratified LGG according to the above risk factors, divided into high-risk groups (tumor recurrence, WHOIII, IDH1 wild, and VASH1 high expression) and low-risk group (primary tumor, WHOII, IDH1 mutation, and VASH1 low expression). By K–M survival curve, the results showed a significantly lower survival time than that of the low-risk group (HR = 8.40, *P*= 0.001), with significant statistical significance (Figure 1(h)).

### 3.7. The Biological Effect of VASH1 on Glioma Cells in Vitro

**Figure 6:**
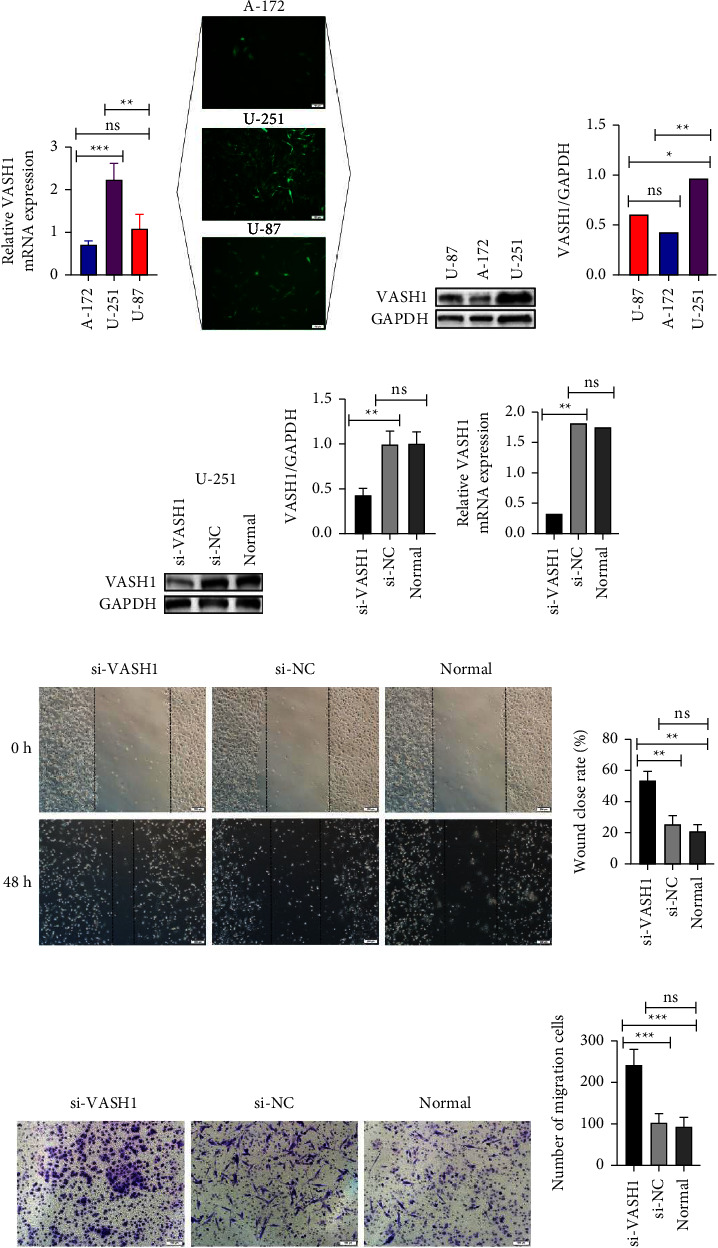
The function of VASH1 was confirmed by in vitro experiments. (a ∼ d) The expression levels of VASH1 in different cell lines were detected by qRT-PCR and western-blot, and the highest expression level was found in the U-251 cell line. (e ∼ f) Western-blot detection of interference efficiency of VASH1 in U-251 cells. (g) Detection of interference efficiency of VASH1 in U-251 cells by qRT-PCR. (h ∼ i) Wound-healing assay indicated that interference of VASH1 expression could promote the migration of U-251 cells. (j ∼ k) Transwell migration assay showed that interference with VASH1 expression promoted the migration ability of U-251 cells. (^*∗*^*P* < 0.05,^*∗∗*^*P* < 0.01, and ^*∗∗∗*^*P* < 0.001).

To further understand the effect of VASH1 expression on the biological behavior of glioma cells, we have constructed VASH1 knockdown glioma cell lines for functional experiments. First, we used real-time PCR and Western-blot to detect the expression of VASH1 mRNA and protein in common human glioma cell lines (A-172, U-251, and U-87) and found that the mRNA and protein expression of VASH1 were highest in the U-251 cell line. Therefore, we planned to select U-251 cells to knockdown VASH1 and conduct subsequent intervention in VASH1 expression experiments and functional experiments (Figures 6(a), 6(b), 6(c), and 6(d)). In this experiment, we transfected VASH1 short hairpin RNA and controlled nonfunctional shRNA plasmid lentivirus (Genechem, Shanghai) with U-251 cells to construct VASH1 knockdown U-251^si−VASH1^, U-251^si−NC^, and its control cell line U-251^Normal^. To determine the expression after infection, the mRNA and protein expression of VASH1 were measured by real-time PCR and Western-blot. The results confirmed that the mRNA and protein expression levels of VASH1 in U-251^si−VASH1^ cells were significantly lower compared with the control and normal U-251 cell line, with a statistically significant difference (*P* < 0.05) (Figures 6(e), 6(f), and 6(g)). The above experiments confirmed that the designed knockout plasmid interference effect was satisfactory, so we performed subsequent functional experiments on the U-251^si−VASH1^, U-251^si−NC^, and U-251^Normal^ cell lines.

The invasion and migration ability of tumor cells are key properties affecting tumor invasion and recurrence, and our previous study confirmed the correlation between VASH1 expression levels and tumor progression. Therefore, we further investigated the effects of VASH1 on the migration and invasion capacity of glioma cells in vitro. First, we used a wound-healing assay to examine the migratory ability of abnormal VASH1 expression on U-251 cells. The results showed that for 48h, U-251^si−VASH1^, U-251^si−NC^, and U-251^Normal^ cells were scratched after the intervening VASH1 expression, the scratches of the U-251^si−VASH1^ healed significantly faster than those of the U-251^si−NC^ and U-251^Normal^ cells (*P* < 0.01) (Figures 6(h) and 6(i)). Next, we performed Tranwell invasion experiments on the above three cell lines, where we seeded U-251^si−VASH1^, U-251^si−NC^, and U-251^Normal^ cells in the upper compartment of the Transwell compartment, adding complete medium and incubating them for 12–24 h. The number of U-251^si−VASH1^ cells passing through the bottom of the Transwell compartment was significantly more than that of the remaining two groups (*P* < 0.001), and no significant difference between the U-251^si−NC^ and U-251^Normal^ cell groups (*P* > 0.05) (Figures 6(j) and 6(k)).

### 3.8. The Biological Function of VASH1 in the LGG

**Figure 7:**
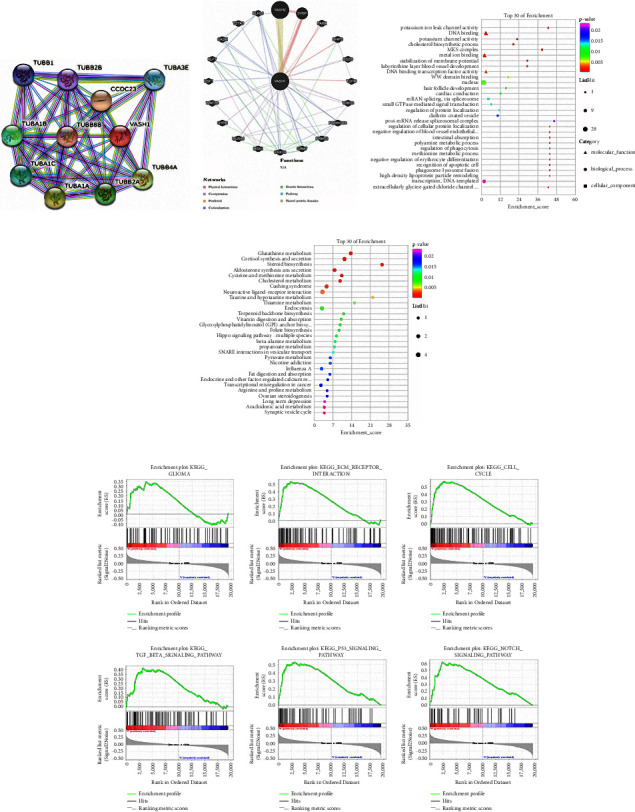
VASH1 gene characteristics and functional analysis. (a) String construction of VASH1 protein co-expression network. (b) GeneMANIA constructed a coexpression network of the VASH1 gene. (c) GO enrichment analysis of VASH1 characteristic gene sets. (d) KEGG enrichment analysis of VASH1-related signaling pathways. (e) GSEA analyzed the correlation between VASH1 and the signaling pathway in the KEGG database.

To further explore the mechanism of VASH1 on gliomas, we first constructed the coexpression network maps of different proteins and genes associated with VASH1 expression through the String and GeneMANIA online database, VASH1 expression was found to be mainly associated with microtubule VASH2, SVBP, PAXX, DHPS, RGS9, TUFM, THBS3, PCDHCC4, PRMT2, GRM7, CXXC4, MSX1, LIRA4, GYP8, WNT2B, ESF1, KLF7, CASK, TSTA3, and KLHL1 (Figures 7(a) and 7(b)). GO analysis found that VASH1 differential genes were mainly enriched in nervous system development, protein binding, structure, and microtubule-binding of the cytoskeleton, DNA repair, and immune cell activation (Figure 7(c)). KEGG analysis showed that VASH1 is associated with cell adhesion factors, Hippo signaling, T cell receptor signaling, endothelial cell migration, and cholesterol synthesis, which indicate that VASH1 is associated with extracellular mechanisms and cellular interactions of the tumor microenvironment (Figure 7(d)). Finally, we initially explored the possible involved signaling pathways through GSEA and showed that VASH1 is mainly related to glioma, ECM receptor, cell cycle, TGF-*β*, P53, and Notch signaling pathway in LGG (Figure 7(e)).

### 3.9. High Expression of VASH1 Was Associated with Immune Infiltration in the LGG

**Figure 8:**
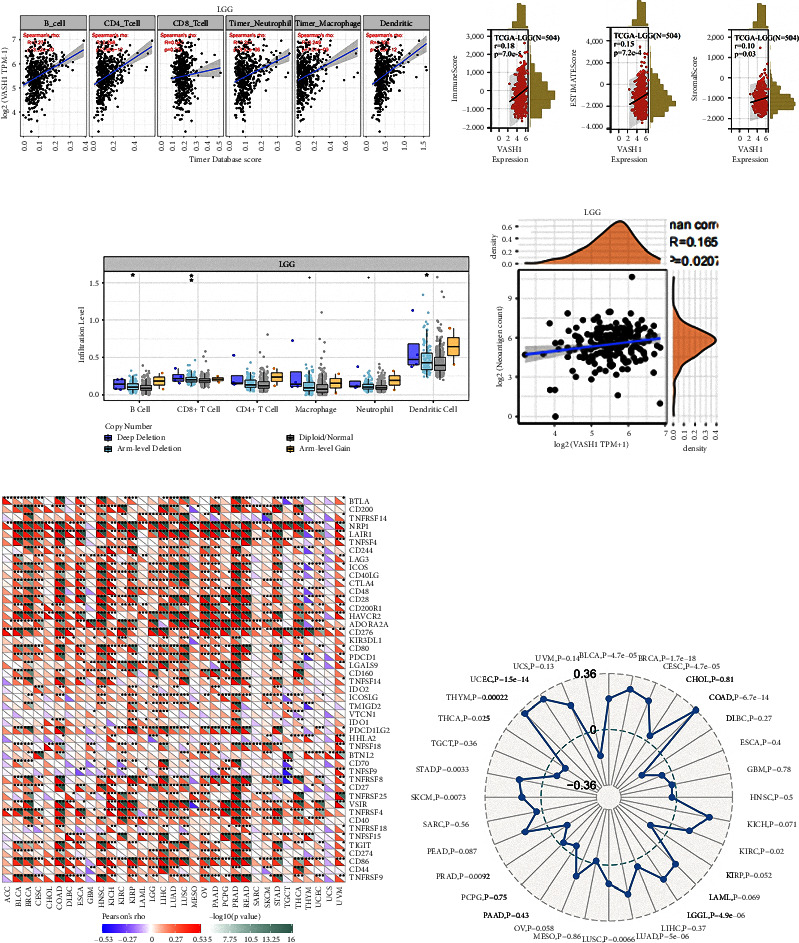
Correlation analysis between VASH1 expression and immune cell infiltration, matrix score, immune checkpoint genes, and TMB of LGG that are closely related. (a) VASH1 expression was positively correlated with immune cell infiltration in LGG. (b) Correlation between VASH1 and ImmuneScore, ESTIMATEScore, and StromalScore in LGG. (c) The correlation between VASH1 expression and somatic copy number alterations examine by TIMER database. (d) VASH1 expression was positively correlated with LGG matrix scores. (e) Correlation analysis between VASH1 expression and 47 immune checkpoint genes in cancer. (f) Correlation analysis between VASH1 expression and TMB in generalized carcinoma. (^*∗*^*P* < 0.05,^*∗∗*^*P* < 0.01, and ^*∗∗∗*^*P* < 0.001).

We analyzed the association of VASH1 on immune infiltration by Timer in the TCGA database. The results found that VASH1 expression was significantly positively associated with the abundance of B cells, CD4^+^T cells, neutrophils, macrophages, and dendritic cells (Figure 8(a)). Next, we investigated the relationship between VASH1 and tumor purity according to the ESTIMATE algorithm. In the LGG, VASH1 expression was positively correlated with matrix score, immune score, and ESTIMATE scores (Figure 8(b)). We also analyzed the value of six types of immune-infiltrating cells in predicting the prognosis of LGG. In addition to macrophages and neutrophils, the infiltration levels of VASH1 of the different copy number types were also different from the other four types of immune-infiltrating cells (*P* > 0.05) (Figure 8(c)). In addition, we used the R package estimate to evaluate the matrix score for each tumor sample, and the VASH1 expression levels were significantly associated with LGG (*R* = 0.165 and *P*=0.020) (Figure 8(d)).

Given that immunotherapy is a key treatment for tumor reduction and eradication, the relationship between VASH1 expression and the expression of the 47 immune checkpoint genes was further analyzed. Interestingly, the analysis indicates that VASH1 expression is positively associated with immune checkpoint-common genes in multiple cancers. In the LGG, VASH1 is associated with CD27. Important relationships exist between CTLA4, CD44, and CD40. This suggests a potential synergy between VASH1 and a consistent immune checkpoint (Figure 8(e)). VASH1 modulates complex patterns of tumor immune responses by regulating immune checkpoint genes. Furthermore, tumor cells with high TMB have high levels of neoantigens that are thought to stimulate the antitumor response of lymphocytes and help the immune system identify tumors. Our analysis showed that VASH1 expression was positively associated with TMB in LGG, THYM, UCEC, BLCA, BRCA, CESC, COAD, LUAD, LUSC, SKCM, and STAD. In contrast, VASH1 expression is inversely correlated with PRAD and THCA (Figure 8(f)). The results further confirmed the interaction between VASH1 and TMB in the LGG.

### 3.10. TME Om characteristics and the Relationship of the LGG Somatic Gene with VASH1

**Figure 9:**
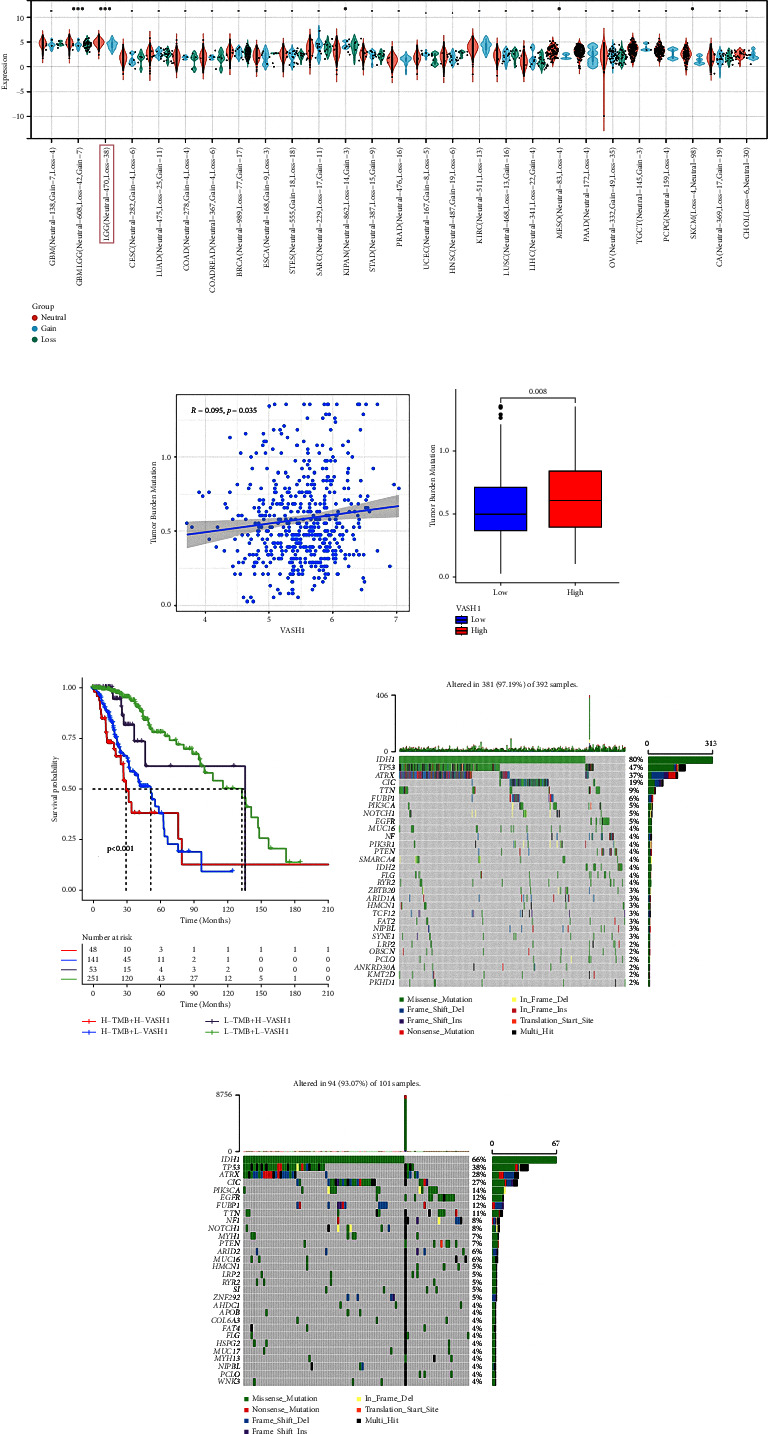
Expression difference of VASH1 gene and somatic variation. (a) VASH1 expression was significantly different in GBM-LGG, LGG, KIPAN, MESO, and SKCM samples. (b) Scatter plot of VASH1 expression related to TMB (P = 0035). (c) Box diagram of different expressions of VASH1 and TMB (*P*=0.008). (d) Kaplan-Meier curves for LGG patients (TCGA-LGG, *P* < 0.001, stratified patients using TMB mutation load, and VASH1 expression). (E ∼ F) are respectively oncoPrint constructed based on VASH1 expression level.

As for indicators of a range of genes or transcriptome fingers related to tumor development, TMB, microsatellite instability, mismatch repair, and methylation are independent risk factors for the efficacy of immune checkpoint blockade (ICB). Therefore, we investigate here the relationship between VASH1 and these indicators to further elucidate whether VASH1 influences LGG occurrence by participating in gene/or transcriptome alteration processes. We calculated the difference in expression of the VASH1 gene invariant and nonvariant samples using *R* software (version 3.6.4), with differential significance analysis using Wilcoxon Rank and Signed Rank Tests. We found significant expression differences in GBM-LGG, LGG, KIPAN, MESO, and SKCM samples (Figure 9(a)). Many studies have shown that TMB can be used to predict the efficacy of ICB and has become a biomarker for various cancer types to identify patients who will benefit from immunotherapy. Based on the important clinical significance of TMB in immunotherapy, we further explored the intrinsic link between TMB and VASH1 expression to elucidate the genetic information related to VASH1. Correlation analysis showed a positive correlation between TMB and VASH1 expression levels (*r* = 0.095 and *P* = 0.035) (Figure 9(b)). In addition, we found the optimal cutoff value through the “survminer” R package based on the expression level of VASH1, divided the patients into high and low expression groups, and performed a box plot visualization, which showed significant differences between the two groups of TMB (*P* = 0.008) (Figure 9(c)). We combined the patient's TMB and VASH1 expression levels for the analysis. The results showed that patients with high VASH1 and the worst prognosis in the high TMB group, while patients in the group with low VASH1 expression and low TMB were the best (*P* = 0.001) (Figure 9(d)). This further suggests a potential association of VASH1 with TMB and suggests that VASH1 and TMB function together and are associated with tumor progression.

In addition, we obtained driver genes for LGG and evaluated somatic mutations in VASH1 patients with different expression levels. Figures 9(e) and 9(f) show the distribution of mutations in the 20 driver genes with the highest frequency of change in the VASH1 high and low groups, respectively, with high VASH1 expression and low VASH1 expression showing a strong correlation with IDH1 and TP53. These results suggest that the combination of VASH1 with IDH1 or TP53 may have implications for LGG risk stratification and guiding therapy.

### 3.11. Correlation Between VASH 1 Expression and Drug Sensitivity

**Figure 10:**
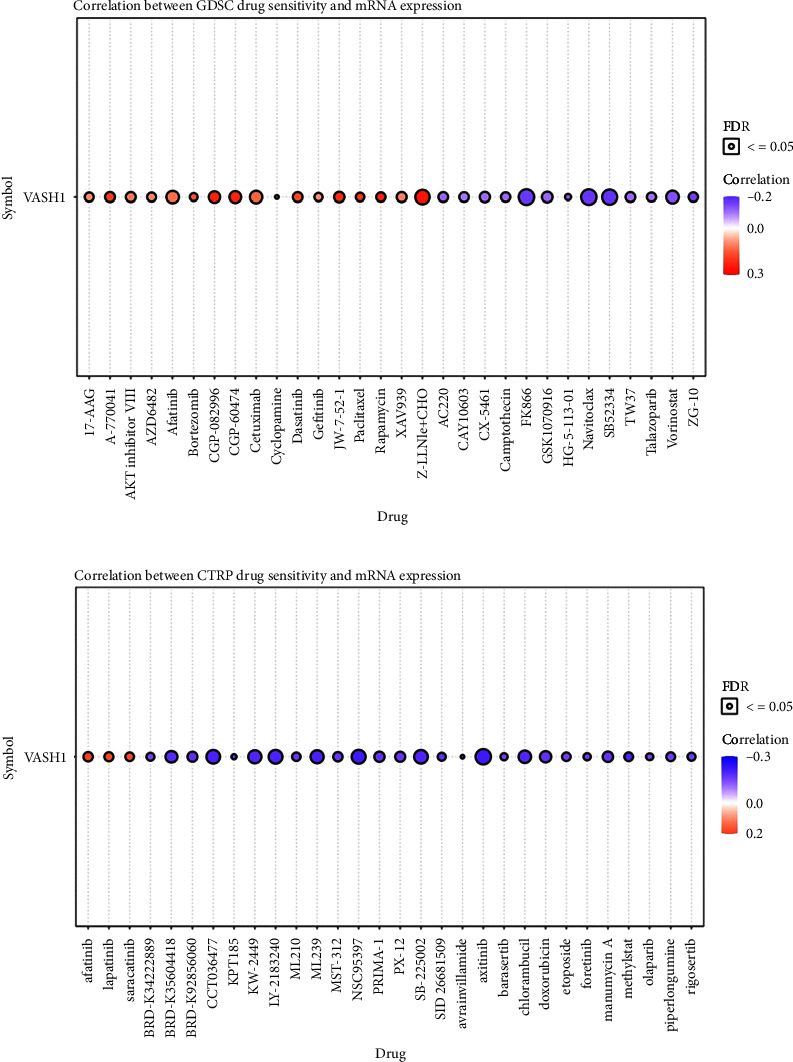
Correlation between drug sensitivity and VASH1 expression. (a and b) The correlation between VASH1 expression and drug sensitivity in pan-RCC was studied by Spearman correlation analysis. Red represents positive correlation between drugs sensitivity and VASH1 expression, and blue represents negative correlation between drugs sensitivity and VASH1 expression. (a): GDSC. (b): CTRP.

Given that VASH1 has potentially promoted LGG progression, it is therapeutically useful to identify anti-LGG drugs targeting VASH1. Therefore, we used GSCA tools to analyze the relationship between VASH1 expression and sensitivity to anti-LGG drugs. VASH1 expression was positively correlated with drug sensitivity for FK866, Navitoclax, SB52334, Z-LLNle-CHO, Vorinostat, Cetuximab, Afatinib, CGP-60474, CGP-082996, GSK1070916, JW-7-52–1, CX-5461, XAV939, A-770041, and AKT inhibitor VIII (all *r* > 0.10 and *P* < 0.01), and negatively associated with drug sensitivity for TW37, AC220, Dasatinib, CAY10603, Camptothecin, ZG-10, Rapamycin, 17-AAG, AZD6482, Genentech Cpd 10, BX-912, Talazoparib, OSI-027, Paclitaxel, and Gefitinib (all *r* < −0.10 and *P* < 0.01)in the Genomics of Drug Sensitivity to Cancer (GDSC) database (Figure 10(a) and Table S1). Similarly, CTRP database analysis demonstrated that VASH1 expression was positively correlated with drug sensitivity for axitinib, NSC95397, and LY-2183240 (all *r* > 0.18, *P* < 0.01), and negatively associated with the drug sensitivity of SB-225002, CCT036477, KW-2449, ML239, chlorambucil, BRD-K35604418, doxorubicin, manumycinA, PRIMA-1, PX-12, BRD-K92856060, afatinib, MST-312, lapatinib, piperlongumine, methylstat, saracatinib, etoposide, ML210, SID26681509, rigosertib, BRD-K34222889, foretinib, barasertib, olaparib, ceranib-2, and ouabain(*r* < −0.15, *P* < 0.01) (Figure 10(b) and Table S2). Taken together, the results demonstrate that VASH1 was correlated with sensitivity to diverse drugs from the Cancer Therapeutic Response Portal database.

## 4. Discussion

Advances in molecular biology in recent years have greatly improved our understanding of diffuse gliomas. At present, IDH1 and 1p 19 q have become genetic indicators of molecular typing of gliomas, which further highlights the importance of molecular typing in the individual diagnosis and treatment of gliomas [21, 22]. As a new vascular regulator derived from endothelial cells, VASH1 can fight against pro-angiogenesis factors and has a negative feedback effect on angiogenesis, and has been found to be related to the occurrence and development of a variety of tumors, becoming a popular “player” in tumor research. A large number of studies have confirmed that VASH1 expression levels are associated with the prognosis of a variety of solid tumors. Yan et al. [23] findings showed that VASH1 expression levels were elevated in colon cancer patients compared with normal colon tissue and significantly positively correlated with VEGF-A, microvessel density (MVD), TNM staging, tumor invasion, lymph node involvement, distant metastasis, and shorter survival. Furthermore, Cox proportional risk regression models showed that VASH1 and lymph node metastasis were independent risk factors affecting the prognosis of colon cancer patients, respectively. Interestingly, in contrast to studies on colon cancer, Zhao [24] et al. showed that high expression of VASH1 was associated with a better prognosis for renal cell carcinoma, and found that its mechanism was related to VASH1's ability to inhibit tumor angiogenesis. This further suggests that VASH1 may play a different role in different tumors [25]. Until now, however, the molecular mechanism of VASH1 in the course of tumorigenesis and development is not entirely clear.

According to studies, in colon cancer, VASH1 is mainly expressed in tumor cells and endothelial cells, and the function of VASH1 is different, and VASH1 overexpression in tumor cells can significantly inhibit the proliferation, migration, and cloning ability of tumor cells [26]. In endothelial cells, VASH1 is transported outside the cell by binding to a chaperone, and then binds to the vascular growth factor receptor-2 (VEGFR-2) on the surface of endothelial cells, inhibiting the activation of the downstream pathway after the binding of VEGF and VEGFR-2, and plays a role in inhibiting angiogenesis [27]. VASH1 inhibits the growth of tumor cells, and overexpression of VASH1 can cause apoptosis (cell death) of dividing cells [28]. For example, in cell cultures for ovarian cancer, VASH1 is able to inhibit insulin-like growth factor 1 (IGF-1) expression and inhibit its angiogenesis [29]. In the kidney cancer model, VASH1 overexpression is able to inhibit tumor growth and promote apoptosis by blocking the cell cycle at the G0/G1 stage. At the same time, VASH1 overexpression can increase tumor sensitivity to chemotherapy [30]. Although higher levels of VASH1 are associated with poorer prognosis for some cancers, this may be due to increased VASH1 feedback in vivo and inhibition of angiogenesis factors (VEGF, FGF-2, and VASH2) in the tumor microenvironment [31]. Miyashita et al. [8] reported that after interfering with the expression of VASH1 in endothelial cells, high expression of VASH1 in addition to inhibiting the activity of endothelial cell tubulation, but also enhance the stress capacity of cells, after low expression of VASH1, cells are easily killed by external stimuli, and suspect that this activity is activated by VASH1 expression of SIRTl and SOD2. By interfering with VASH1 expression with lentivirus, they found that after VASH1 knockdown, endothelial cells develop autophagy and premature aging, and endothelial cells are very susceptible to death due to external stimuli.

Therefore, it is explained that VASH1 regulates the activity of tumor cells and endothelial cells through multiple signaling pathways in different tissues. In addition, immunostaining of postoperative specimens of patients with esophageal cancer by Ninomiya [32] et al. found that VASH1 and VASH2 expression were associated with tumor progression and prognosis, with VASH1-positive patients with esophageal cancer having a poor prognosis. However, due to the lack of typical secretory signaling sequences of these two regulators, VASH1 needs to bind to small vasohibin-binding protein (SVBP) to function, and promote the expression of *α*-tubulin (*α* -tubulin), increase the stability of tumor cell structure, and further inhibit tumor growth leading to tumor progression. Nieuwenhuis et al. [27] found that after VASH1 binds to SVBP in the nervous system, microtubule production is regulated, thereby improving the stability of nerve cells. Conversely, a decrease in VASH1 expression leads to cell carcinogenesis or neurodevelopmental abnormalities. However, the mechanism of action of VASH1 in gliomas has not been elucidated.

To our knowledge, the present study is the first to comprehensively evaluate VASH1 expression and its association with clinical and prognostic outcomes in LGG using various public databases, including the CGGA, TCGA, GPEIA2, and ULCAN datasets. We found that VASH1 was significantly overexpressed in LGG and that increased VASH1 expression correlated significantly with poor outcomes, WHO grade, IDH mutation status, and recurrence. Cox and univariate analyses showed that high expression of VASH1, WHOIII, recurrence and IDH1 wild type were independent risk factors for the prognosis of LGG patients. Based on multivariate Cox analysis, a nomogram was constructed to predict the prognosis of patients with LGG the expression of VASH1 and to stratify LGG patients with better performance.

Secondly, in glioma, current studies recognize that IDH1 mutations are closely associated with glioma grade and prognosis, however, there is a lack of molecules associated with IDH1 [22]. We first found, by analyzing the TCGA database of VASH1 and LGG that VASH1 was closely associated with prognosis not only in LGG patients (*P* < 0.05). However, analysis of the CCGA database revealed no significant correlation between VASH1 and IDH1 in LGG (*P*=0.24). Therefore, to further validate this, we performed immunohistochemical staining of tissue samples from patients with different grades of LGG and followed up the patients. The results showed that both VASH1 and IDH1 were independent predictors of prognosis in LGG patients, and high VASH1 expression was significantly correlated with IDH1 wild-type status, while further reflecting tumor progression and recurrence status. We speculate that the mechanism of action of VASH1 in glioma may have some correlation with IDH1 and may provide a potential molecular marker for risk stratification of high-risk gliomas.

In order to further explore the biological role of VASH1 in glioma cells, we first detected VASH1 expression in three human glioma cell lines (A-172, U-251, and U-87) and screened according to the results by Western-Blot and qPCR, and then established the human glioma cell line knocked out by VASH1 and analyzed the effect of low expression of VASH1 on the biological activity of glioma cells. According to Zhao et al. [24], VASH1 overexpression can inhibit the proliferation of human umbilical cord endothelial cells and 786–0 cells, promote apoptosis, but cannot inhibit tumor invasion. Fu et al. [33] have shown that inhibition of VASH1 expression can significantly promote the proliferation, migration, and invasion of U-87 cells. It was further determined that the biological role of VASH1 in different tissues is different. Our findings show that VASH1 expression is highest in U-251 cell lines, and the ability to inhibit the migration and invasion of U-251-expressing cells is significantly increased, which is consistent with the results of Fu et al. In summary, we found that inhibiting VASH1 expression in in vitro experiments promotes tumor cell line migration and invasion, while in contrast to human LGG patients, high VASH1 expression is associated with poor prognosis, so we explored the mechanism of action of VASH1 in LGG through GO, KEGG, and GSEA.

The results of the GO and KEGG enrichment analysis of this study show that VASH1 is mainly closely related to cytoskeleton, microtubule construction, Hippo signaling pathway, T cell receptor signaling pathway, and endothelial cell migration. By constructing the gene and protein PPI map, it was found that VASH1 was mainly associated with microtubule-associated proteins in LGG, which was consistent with the Nieuwenhuis [27] report that VASH1 mutants specifically led to abnormal tyrosineization and de-tyrosylation dynamic circulation of *α*-tubulin, which was closely related to cell transformation and glioma invasion. GSEA enrichment analysis further revealed that the role of VASH1 in glioma may be correlated by cell cycle, P53, Notch, and TGF-*β* signaling pathways. It was found that microtubules play an important role in mitogenic spindle formation, cell morphology maintenance, intracellular substance transport, immune cell infiltration, and chromosomal isolation, and can affect the proliferation of glioma cells. Interestingly, VASH1-SVBP plays a regulatory role in *α*-tubulin tyrosineization and induces the ossification of microtubulins by the polymerization of tubulins, a morphological alteration associated with reduced invasion of gliomas. In summary, from the above results, we believe that VASH1 plays an antitumor role as an endogenous antitumor factor in LGG, and the feedback increase of VASH1 expression plays a variety of roles during tumor progression. On the one hand, it reflects the state of tumor progression. On the other hand, as an endogenous antitumor factor, it acts on tumor cells and endothelial cells to inhibit the growth of tumors. However, since in vitro tests cannot mimic the tumor microenvironment in vivo, VASH1 only acts on tumor cells, inhibiting the decline in stability of tumor cells after its expression, thereby promoting the proliferation ability of tumors. Ma et al [31]. have come to similar conclusions in esophageal cancer, suggesting that VASH1 has a special biological role and may be a potential target for tumor therapy. This result requires more in vivo and in vitro studies to further validate.

In addition, we also found that VASH1 expression has a certain correlation with the immune cell response signaling pathway, and through immunohistochemical staining, we found that VASH1 is not only expressed in tumor cells and endothelial cells, but also in small quantities in lymphocytes. Tumor infiltrating immune cells are closely related to tumorigenesis, angiogenesis, and tumor cell growth, thereby regulating the number and differentiation of immune cells [34]. There is evidence that tumor progression may be due to cancer cells escaping from host immune monitoring [35]. Therefore, elucidating the molecular mechanisms that may be involved in infiltrating immune cells in TME could provide new immune targets for LGG. We found that the proportion of antitumor immune cells was higher in the VASH1 high-expression group, and that VASH1 expression was positively correlated with infiltration of B cells, CD4+ T cells, macrophages, neutrophils, and dendritic cells. In addition, we also found that VASH1-mediated differential genes and rich immune pathways, VASH1 is positively correlated with KLHL-1, PAXX, and CXXC4 in LGG, and is associated with natural killer cell-mediated cytotoxic pathways, thereby mediating NK cell and macrophage infiltration, which plays an important role in antitumor immune response. At the same time, we found that VASH1 has a clear correlation with TMB in LGG, so we believe that VASH1 can be used as a potential immunotherapy target and reflect the ability and extent of tumor production of neoantigens, predicting the immunotherapy efficacy of LGG.

Most endogenous factors are associated with a better prognosis, however, the opposite is true for VASH1. This is the interesting point of our paper, as tumor growth and progression are influenced by multiple factors, including genetic alterations, remodeling of the microenvironment, and suppression of immune cells. VASH1, on the other hand, acts as an endogenous factor, and its expression feedback line increases during tumor progression, inhibiting tumor growth to some extent, however, because tumor progression is a complex process, its inhibitory effect is limited, resulting in VASH1 only as a biomarker reflecting the role of tumor progression, while its actual inhibitory effect in the microenvironment is limited. In in vitro experiments, since it is impossible to mimic the tumor microenvironment, where alteration of certain factors can directly affect tumor proliferation and growth, VASH1 acts as its endogenous antitumor effect in vitro. Also, VASH1 in esophageal cancer, the exact mechanism of which needs to be followed up with continued research [27, 31]. This study may provide a new idea for the diagnosis and treatment of lower-grade gliomas.

This study had several limitations. Although we confirmed the effect of knockout VASH1 on the promotion of glioma U-251 cell lines through in vitro experiments, its expression in the rest of the glioma cell lines was low, and its knockout VASH expression was found to be poor by qRT-PCR and Western-Blot detection, considering the need to further confirm this result due to our small VASH1 sequence. In addition, we explored its mechanism of action in gliomas through GO, KEGG, and GSEA, but this was not confirmed by in vivo and in vitro tests. Furthermore, the present study assessed the expression and biological roles of VASH1 in databases of patients with LGG and cultured cells, not in vivo. Additional studies are required to assess the function of VASH1 in LGG progress and in regulating the glioma tumor microenvironment.

## 5. Conclusion

Based on the above results, we found that VASH1 was highly expressed in LGG, and the high expression of VASH1 was closely related to the poor prognosis of LGG patients. At the same time, bioinformatics and experiments confirmed the high specificity and biological characteristics of VASH1 in LGG, and preliminarily clarified that VASH1 acts as an endogenous antitumor factor in LGG and plays a dual role in the increase of VASH1 expression feedback in the progression of LGG. It mainly reflects the progress of tumor and inhibits tumor growth by acting on tumor cells, immune cells and endothelial cells. In conclusion, this study helps us to better understand the biological characteristics of VASH1 in LGG, and provides a new idea for individualized treatment of LGG patients.

## Figures and Tables

**Table 1:** Tumor name and abbreviations.

Abbreviations	Tumor name
ACC	Adrenocortical Carcinoma
BLCA	bladder Urothelial Carcinoma
BRCA	Breast Invasive Carcinoma
CESC	Cervical Squamous Cell Carcinoma And Endocervical Adenocarcinoma
CHOL	Cholangiocarcinoma
COAD	colon Adenocarcinoma
DLBC	Lymphoid Neoplasm Diffuse Large B-Cell Lymphoma
ESCA	Esophageal Carcinoma
HNSC	Head and Neck Squamous Cell Carcinoma
KICH	Kidney chromophobe
KIRC	Kidney Renal Clear Cell Carcinoma
KIRP	Kidney Renal Papillary Cell Carcinoma
LAML	Acute Myeloid Leukemia
LGG	Lower-Grade Glioma
LIHC	Liver Hepatocellular Carcinoma
LUAD	Lung Adenocarcinoma
LUSC	Lung Squamous Cell Carcinoma
MESO	Mesothelioma
OV	Ovarian Serous Cystadenocarcinoma
PAAD	Pancreatic Adenocarcinoma
PCPG	Pheochromocytoma and Paraganglioma
PRAD	Prostate Adenocarcinoma
READ	Rectum Adenocarcinoma
SARC	Sarcoma
SKCM	Skin Cutaneous Melanoma
STAD	Stomach Adenocarcinoma
TGCT	Testicular Germ Cell Tumors
THCA	Thyroid Carcinoma
UCEC	Uterine Corpus Endometrial Carcinoma
UCS	Uterine Carcinosarcoma
UVM	Uveal Melanoma

**Table 2:** Correlations between VASH1 expression and clinicopathological characteristics in TCGA-LGG patients.

Characteristic	Low expression of VASH1	High expression of VASH1	*p*
*n*	264	264	

WHO grade, *n* (%)			<0.001
G2	135 (28.9%)	89 (19.1%)	
G3	97 (20.8%)	146 (31.3%)	

IDH status, *n* (%)			0.026
WT	38 (7.2%)	59 (11.2%)	
Mut	224 (42.7%)	204 (38.9%)	

1p/19q codeletion, *n* (%)			0.457
Codel	81 (15.3%)	90 (17%)	

Non-codel	183 (34.7%)	174 (33%)	

Gender, *n* (%)			0.861
Female	121 (22.9%)	118 (22.3%)	
Male	143 (27.1%)	146 (27.7%)	

Age, *n* (%)			0.931
≤40	131 (24.8%)	133 (25.2%)	
>40	133 (25.2%)	131 (24.8%)	

Histological type, *n* (%)			0.485
Astrocytoma	95 (18%)	100 (18.9%)	
Oligoastrocytoma	73 (13.8%)	61 (11.6%)	
Oligodendroglioma	96 (18.2%)	103 (19.5%)	

Primary therapy outcome, *n* (%)			0.055
PD	46 (10%)	64 (14%)	
SD	69 (15.1%)	77 (16.8%)	
PR	33 (7.2%)	31 (6.8%)	
CR	81 (17.7%)	57 (12.4%)	

**Table 3:** Univariate and multivariate analysis for the VASH1 risk score and overall survival in different LGG patient cohorts.

Characteristics	Total (*N*)	Univariate analysis		Multivariate analysis	
		Hazard ratio (95% CI)	*P* Value	Hazard ratio (95% CI)	*P* Value
*TCGA cohort*					
WHO grade	466				
G2	223				
G3	243	3.059 (2.046–4.573)	<0.001	1.914 (1.148–3.191)	0.013
1p19q_codeletion_status	527				
Codel	170				
Non-codel	357	2.493 (1.590–3.910)	<0.001	0.992 (0.503–1.958)	0.982
Primary therapy outcome	256				
SD	146				
PD	110	2.288 (1.520–3.446)	<0.001	2.578 (1.548–4.295)	<0.001
IDH status	524				
WT	97				
Mut	427	0.186 (0.130–0.265)	<0.001	0.418 (0.235–0.744)	0.003
Age	527				
≤40	264				
>40	263	2.889 (2.009–4.155)	<0.001	3.499 (2.124–5.763)	<0.001
Histological type	527				
Astrocytoma	195				
Oligoastrocytoma	134	0.661 (0.421–1.037)	0.071	1.774 (1.012–3.110)	0.045
Oligodendroglioma	198	0.577 (0.392–0.848)	0.005	0.560 (0.306–1.024)	0.060
VASH1	527				
Low	264				
High	263	1.557 (1.107–2.192)	0.011	0.914 (0.564–1.482)	0.717

*CGGA cohort*					
Grade	544				
G2	250				
G3	294	3.100 (2.381–4.037)	<0.001	2.670 (2.013–3.542)	<0.001
1p19q_codeletion_status	506				
Noncodel	347				
Codel	159	0.287 (0.206–0.399)	<0.001	0.333 (0.236–0.471)	<0.001
PRS_type	544				
Recurrent	176				
Primary	368	0.363 (0.285–0.464)	<0.001	0.408 (0.315–0.528)	<0.001
IDH_mutation_status	544				
WT	137				
Mut	407	0.442 (0.343–0.570)	<0.001	0.616 (0.467–0.813)	<0.001
Age	544				
>40	263				
≤40	281	0.847 (0.668–1.075)	0.173		
Gender	544				
Female	232				
Male	312	0.915 (0.719–1.164)	0.468		
VASH1	544				
Low	266				
High	278	1.571 (1.233–2.002)	<0.001	1.492 (1.151–1.933)	0.002

**Table 4:** Correlations between VASH1 expression and clinicopathological characteristics in LGG patients.

Parameters	VASH1 expression		t/*χ*^2^	*P -*value
	High expression (*n* = 53)	Low expression (*n* = 30)		
Age (year)	51.5 ± 15.6	48.5 ± 16.3	0.869	0.387

Gender			0.044	0.834
Male	27 (32.5%)	16 (19.3%)		
Female	26 (31.3%)	14 (16.9%)		

Histology			1.163	0.559
Astrocytomas	34 (41.0%)	21 (25.3%)		
Oligodendrogliomas	14 (16.9%)	5 (6.0%)		
Oligoastrocytoma	5 (6.2%)	4 (4.8%)		

Relapse			15.256	<0.001
Primary	17 (20.5%)	23 (27.8%)		
Recurrence	36 (43.4%)	7 (8.4%)		

Tumor diameter			1.182	0.277
<4 cm	20 (24.1%)	15 (18.1%)		
≥4 cm	33 (39.8%)	15 (18.1%)		

Tumor location			0.967	0.616
Frontal lobe	20 (24.1%)	14 (16.9%)		
Temporal lobe	22 (26.5%)	12 (14.5%)		
Other	11 (13.3%)	4 (4.8%)		

WHO stage			10.592	0.001
II	27 (31.9%)	26 (23.4%)		
III	26 (25.5%)	4 (7.4%)		

Karnofsky (KPS)			0243	0.622
<70	15 (18.1%)	7 (8.4%)		
≥70	38 (45.8%)	23 (27.7%)		

Epilepsy			2.623	0.105
Yes	31 (37.2%)	12 (10.6%)		
No	22 (23.4%)	18 (28.7%)		

IDH1 type			11.893	0.001
Wild	35 (42.2%)	8 (9.6%)		
Mutant	18 (21.7%)	22 (26.5%)		

**Table 5:** Univariate analysis of the prognostic factors and survival of our patient with LGG.

Parameters	Number of patients	Deaths	Survivals	Log-rank*χ*^2^	*P -*value
Age (year)					

Gender				0.268	0.605
Male	43	23	20		
Female	40	20	17		

Histology				1.120	0.571
Astrocytomas	55	32	23		
Oligodendrogliomas	19	9	10		
Ependymomas	9	5	4		

Relapse				31.787	<0.001
Primary	40	17	23		
Recurrence	43	29	14		

Tumor diameter				0.990	0.320
<4 cm	35	23	12		
≥4 cm	48	23	25		

Tumor location				0.648	0.723
Frontal lobe	34	21	13		
Temporal lobe	34	18	16		
Other	15	7	8		

WHO stage				36.438	<0.001
II	52	28	24		
III	31	18	13		

Karnofsky (KPS)				1.178	0.180
<70	22	15	7		
≥70	61	31	30		

Epilepsy				3.411	<0.001
Yes	43	25	18		
No	40	21	19		

IDH1 type				38.974	<0.001
Wild	43	25	18		
Mutant	40	21	19		

VASH1 expression				20.615	<0.001
High	53	30	23		
Low	30	16	14		

**Table 6:** Multivariate Cox analyses of OS in our LGG patients.

Parameters	HR	95%CI Low	95%CI High	*P*-value
Relapse	2.375	1.096	5.147	0.028
IDH1 type	6.473	2.601	16.109	<0.001
WHO garde	2.679	1.092	6.570	0.031
VASH1	4.753	1.768	12.776	0.002

## Data Availability

The datasets used and/or analyzed during the current study are available from the corresponding author.

## Supplementary Materials

Supplementary Materials Supplementary Table 1: Analysis of the correlation between VASH1 expression and diverse drug sensitivity by GDCS database; Supplementary Table 2: Analysis of the correlation between VASH1 expression and diverse drug sensitivity by CTRP database. Original images: Original images (Transwell) and original images (western-blot). Original data: Original data(PCR).
