# Supplementary material for: Identification of VASH1 as a Potential Prognostic Biomarker of Lower-Grade Glioma by Quantitative Proteomics and Experimental Verification
Source: J Oncol. 2022 Nov 30;2022:2621969. doi: 10.1155/2022/2621969 (PMC9729035; doi:10.1155/2022/2621969)
Supplement: Supplementary Materials — Supplementary Table 1: Analysis of the correlation between VASH1 expression and diverse drug sensitivity by GDCS database; Supplementary Table 2: Analysis of the correlation between VASH1 expression and diverse drug sensitivity by CTRP database. Original images: Original images (Transwell) and original images (western-blot). Original data: Original data(PCR). [file 2621969.f1.zip › (Supplementary)Table.S2.docx]

| **Symbol** | **Drug** | **Cor** | **FDR** |
| --- | --- | --- | --- |
| VASH1 | axitinib | 0.19 | 4.38E-09 |
| VASH1 | NSC95397 | 0.18 | 4.62E-08 |
| VASH1 | LY-2183240 | 0.18 | 5.69E-08 |
| VASH1 | SB-225002 | -0.15 | 6.74E-08 |
| VASH1 | CCT036477 | -0.15 | 6.91E-08 |
| VASH1 | KW-2449 | -0.16 | 1.39E-07 |
| VASH1 | ML239 | -0.16 | 1.52E-07 |
| VASH1 | chlorambucil | -0.16 | 3.28E-07 |
| VASH1 | BRD-K35604418 | -0.16 | 1.12E-06 |
| VASH1 | doxorubicin | -0.16 | 1.36E-06 |
| VASH1 | manumycin A | -0.16 | 3.54E-06 |
| VASH1 | PRIMA-1 | -0.16 | 4.03E-06 |
| VASH1 | PX-12 | -0.17 | 4.88E-06 |
| VASH1 | BRD-K92856060 | -0.17 | 4.99E-06 |
| VASH1 | afatinib | -0.17 | 8.75E-06 |
| VASH1 | MST-312 | -0.17 | 8.91E-06 |
| VASH1 | lapatinib | -0.18 | 1.11E-05 |
| VASH1 | piperlongumine | -0.18 | 1.35E-05 |
| VASH1 | methylstat | -0.18 | 1.41E-05 |
| VASH1 | saracatinib | -0.18 | 1.57E-05 |
| VASH1 | etoposide | -0.19 | 1.63E-05 |
| VASH1 | ML210 | -0.19 | 1.70E-05 |
| VASH1 | SID 26681509 | -0.2 | 2.24E-05 |
| VASH1 | rigosertib | -0.2 | 2.31E-05 |
| VASH1 | BRD-K34222889 | -0.2 | 2.38E-05 |
| VASH1 | foretinib | -0.2 | 3.56E-05 |
| VASH1 | barasertib | -0.2 | 3.72E-05 |
| VASH1 | olaparib | -0.21 | 4.48E-05 |
| VASH1 | ceranib-2 | -0.22 | 4.69E-05 |
| VASH1 | ouabain | -0.22 | 4.86E-05 |

****Table S2****. Analysis of the correlation between BIRC5 expression and diverse drug sensitivity by CTRP database.
