# Supplementary material for: Identification of VASH1 as a Potential Prognostic Biomarker of Lower-Grade Glioma by Quantitative Proteomics and Experimental Verification
Source: J Oncol. 2022 Nov 30;2022:2621969. doi: 10.1155/2022/2621969 (PMC9729035; doi:10.1155/2022/2621969)
Supplement: Supplementary Materials — Supplementary Table 1: Analysis of the correlation between VASH1 expression and diverse drug sensitivity by GDCS database; Supplementary Table 2: Analysis of the correlation between VASH1 expression and diverse drug sensitivity by CTRP database. Original images: Original images (Transwell) and original images (western-blot). Original data: Original data(PCR). [file 2621969.f1.zip › Supplementary(Table.S1).docx]

****Table S1****. Analysis of the correlation between BIRC5 expression and diverse drug sensitivity by GDCS database.

| **Symbol** | **Drug** | **Cor** | **FDR** |
| --- | --- | --- | --- |
| VASH1 | FK866 | 0.26 | 2.07E-06 |
| VASH1 | Navitoclax | 0.24 | 2.42E-06 |
| VASH1 | SB52334 | 0.23 | 4.55E-06 |
| VASH1 | Z-LLNle-CHO | 0.23 | 1.34E-05 |
| VASH1 | Vorinostat | 0.22 | 1.52E-04 |
| VASH1 | Cetuximab | 0.2 | 2.00E-04 |
| VASH1 | Afatinib | 0.2 | 2.82E-04 |
| VASH1 | CGP-60474 | 0.18 | 4.49E-04 |
| VASH1 | CGP-082996 | 0.14 | 6.55E-04 |
| VASH1 | GSK1070916 | 0.13 | 1.54E-03 |
| VASH1 | JW-7-52-1 | 0.12 | 1.97E-03 |
| VASH1 | CX-5461 | 0.11 | 2.26E-03 |
| VASH1 | XAV939 | 0.11 | 3.01E-03 |
| VASH1 | A-770041 | 0.1 | 3.05E-03 |
| VASH1 | AKT inhibitor VIII | 0.1 | 3.11E-03 |
| VASH1 | TW 37 | -0.1 | 3.50E-03 |
| VASH1 | AC220 | -0.1 | 3.59E-03 |
| VASH1 | Dasatinib | -0.1 | 3.63E-03 |
| VASH1 | CAY10603 | -0.1 | 4.14E-03 |
| VASH1 | Camptothecin | -0.11 | 4.89E-03 |
| VASH1 | ZG-10 | -0.11 | 5.01E-03 |
| VASH1 | Rapamycin | -0.11 | 5.15E-03 |
| VASH1 | 17-AAG | -0.11 | 6.51E-03 |
| VASH1 | AZD6482 | -0.12 | 6.70E-03 |
| VASH1 | Genentech Cpd 10 | -0.12 | 6.74E-03 |
| VASH1 | BX-912 | -0.14 | 6.81E-03 |
| VASH1 | Talazoparib | -0.16 | 7.70E-03 |
| VASH1 | OSI-027 | -0.17 | 7.91E-03 |
| VASH1 | Paclitaxel | -0.17 | 9.03E-03 |
| VASH1 | Gefitinib | -0.17 | 0.01 |
